# Effects of acute exercise on circulating endothelial and progenitor cells in children and adolescents with juvenile idiopathic arthritis and healthy controls: a pilot study

**DOI:** 10.1186/s12969-015-0038-4

**Published:** 2015-10-12

**Authors:** Joyce Obeid, Thanh Nguyen, Tania Cellucci, Maggie J. Larché, Brian W. Timmons

**Affiliations:** Child Health and Exercise Medicine Program, McMaster University, HSC 3N27G, 1280 Main Street West, Hamilton, L8S 4K1 ON Canada; Department of Pediatrics, Division of Rheumatology, McMaster University, 1280 Main St W, Health Sciences Centre, Hamilton, L8S 4K1 ON Canada; Departments of Medicine and Pediatrics, Division of Rheumatology, McMaster University, Hamilton, ON Canada; Department of Pediatrics, McMaster University, 1280 Main St W, Health Sciences Centre, Hamilton, L8S 4K1 ON Canada; Department of Medicine, Charlton Medical Centre, 25 Charlton Ave E, Suite 702, Hamilton, L8N1Y2 ON Canada

**Keywords:** Juvenile idiopathic arthritis, Flow cytometry, Exercise, Cardiovascular risk factors, Endothelial progenitor cells, Circulating endothelial cells

## Abstract

**Background:**

Youth with juvenile idiopathic arthritis (JIA) may be at risk of poor cardiovascular health. Circulating endothelial progenitor cells (EPCs) and circulating endothelial cells (CECs) are markers of cardiovascular repair and damage, respectively, and respond to exercise. The objectives of this study were to compare resting levels of EPCs and CECs in JIA and controls, and to assess the effects of distinct types of exercise on EPCs and CECs in JIA and controls.

**Methods:**

Seven youth with JIA and six controls completed 3 visits. First, aerobic fitness was assessed. Participants then performed either moderate intensity, continuous exercise (MICE) or high intensity, intermittent exercise (HIIE) on separate days. Blood samples were collected at the beginning (REST), mid-point (MID) and end of exercise (POST) for determination of EPCs (CD31^+^CD34^bright^CD45^dim^CD133^+^) and CECs (CD31^bright^CD34^+^CD45^−^CD133^−^) by flow cytometry. Between group differences in EPCs and CECs were examined using two-way ANOVA, followed by Tukey’s HSD post hoc, where appropriate. Statistical significance set at p ≤ 0.05.

**Results:**

Both EPCs and CECs were similar between groups at REST (p = 0.18–0.94). During MICE, EPCs remained unchanged in JIA (p = 0.95) but increased significantly at POST in controls (REST: 0.91 ± 0.55 × 10^6^ cells/L vs. POST: 1.53 ± 0.36 × 10^6^ cells/L, p = 0.04). Compared with controls, lower levels of EPCs were observed in JIA at MID (0.48 ± 0.50 × 10^6^ cells/L vs. 1.10 ± 0.39 × 10^6^ cells/L, p = 0.01) and POST (0.38 ± 0.34 × 10^6^ cells/L vs. 1.53 ± 0.36 × 10^6^ cells/L, p < 0.001) during MICE. No changes were detected in CECs with MICE in JIA and controls (p = 0.69). Neither EPCs nor CECs were modified with HIIE (p = 0.28–0.69).

**Conclusion:**

Youth with JIA demonstrated a blunted EPC response to MICE when compared with controls. Future work should examine factors that may increase or normalize EPC mobilization in JIA.

## Background

Children and adolescents with juvenile idiopathic arthritis (JIA) may be at an increased risk of developing poor cardiovascular health, with early signs of atherosclerosis manifesting as young as 4 years of age [1, 2]. This finding might be expected given that cardiovascular disease (CVD) is the leading cause of death among adults with rheumatoid arthritis (RA), and a number of predisposing factors for CVD, including chronic inflammation and low levels of physical activity, are also present on a long-term basis in JIA [3–6]. In fact, recent evidence suggests that approximately 41 % of patients with JIA maintain an active disease state, marked by increased levels of inflammation and medication usage, 30 years after disease onset [7]. Interestingly, the increased incidence of CVD in adults with RA cannot be solely explained by traditional risk factors, highlighting the need for sensitive and specific alternative markers of CV health in this population and in younger patients with JIA [3, 8].

Recent evidence from healthy controls and patients supports a critical role for two distinct cell populations, circulating endothelial progenitor cells and circulating endothelial cells, in CV health [9, 10]. Circulating endothelial progenitor cells (EPCs) are a heterogeneous population of cells derived from the bone marrow that contribute to vascular repair and post-natal vasculogenesis via paracrine secretion of angiogenic factors or differentiation into mature endothelial cells [11, 12]. Concentrations of EPCs are inversely associated with markers of endothelial dysfunction and are known to predict occurrence of cardiovascular events [9, 13]. Moreover, EPCs are reduced in numerous pathologic conditions, including RA [13–17]. Much less is known about circulating endothelial cells (CECs), which represent a population of mature endothelial cells shed from the intima following irreversible structural damage to the endothelium [18]. Elevated levels of CECs are consistently reported in individuals with known risk factors for cardiovascular disease, including RA, and may be predictive of cardiovascular events [10, 13, 19]. Only one study has assessed EPCs in children with JIA and reported similar concentrations to healthy controls [20]. Conversely, CECs have yet to be assessed in JIA; however, the available evidence in various clinical populations suggest that both EPCs and CECs cells may provide important, clinically relevant insight into CV repair and damage, respectively.

Given the altered EPC and CEC profiles observed in clinical populations at risk of CVD, identification of methods to enhance their mobilization may allow for the development of therapeutic agents to improve CV remodeling. Exercise may represent one such stimulus as it has been shown to increase peripheral blood EPCs in adults and children [21–25]. Specifically, acute bouts of both high and moderate intensity running in adults led to similar increases in EPCs of 34–120 % and 20–163 %, respectively [22]. In healthy children, EPCs have only been examined in response to an acute bout of high intensity intermittent cycling, which increased EPCs by 83–170 % [23]. Unlike EPCs, the effect of acute exercise on CECs has only been examined in two studies of older adults with CVD with mixed results; however, a negative association between CECs and habitual physical activity among healthy children was recently reported [26–28]. Thus, exercise-induced changes in EPCs and CECs require additional study as one potential mechanism to enhance CV repair and remodeling, and ultimately impact overall CV health.

To date, no study has examined both resting and exercise-related changes in EPCs and CECs in JIA or compared the effects of distinct types of exercise on these cells. As such, the objectives of this study were to: 1) compare resting levels of EPCs and CECs in youth with JIA and healthy, age-matched controls; 2) assess the effects of acute bouts of high intensity and moderate intensity exercise on EPCs and CECs in JIA; and 3) compare exercise-induced changes in EPCs and CECs in JIA and healthy controls. Given the high levels of inflammation and low levels of physical activity and fitness reported in JIA, it is plausible that they would demonstrate lower levels of EPCs and elevated CECs at rest compared with their healthy peers [6, 29]. Further, we hypothesized that specific episodes of exercise, regardless of intensity, would lead to a transient increase in EPCs in both JIA and controls. Since CECs are negatively associated with CV health, and exercise is a potent stimulus to improve CV health, we hypothesized that acute exercise may transiently decrease CECs [18, 30, 31].

## Methods

Children with JIA were recruited by a research coordinator during their regularly scheduled visit to the McMaster Children’s Hospital Pediatric Rheumatology Clinic. All patients were 8 to 17 years of age, and were diagnosed with JIA in accordance with the International League of Associations for Rheumatology criteria [32]. Patients were only excluded from participation if they did not have a confirmed JIA diagnosis, were diagnosed with any other medical condition, or had contraindications for exercise, including joint pain or swelling that would prevent completion of the exercise tests. They were also excluded if they were currently on biologic therapy or had received corticosteroid injections within 3 months of study participation as these treatments may alter resting EPCs or their precursors [20, 33, 34]. Healthy controls were matched by age to participants with JIA (within 1 year) and were either friends of participants with JIA or were recruited from the general community. Participants were not matched by sex as our previous work revealed no sex differences in either EPCs or CECs [26]. Healthy participants were excluded if they had any known medical conditions or had a BMI ≥ 85^th^ percentile for their age, since these might impact EPCs and CECs. All participants and parents/guardians provided written informed consent and assent, respectively, prior to enrollment in this study, which was approved by the Hamilton Health Sciences/Faculty of Health Sciences Research Ethics Board (REB #08-276).

### Study overview

Demographic and disease-related characteristics were retrieved by retrospective review of each participant’s health records. All participants were invited to the Child Health & Exercise Medicine Program research laboratory on 3 occasions. During their first visit, basic anthropometric data were collected, including standing height measured to the nearest 0.1 cm, and weight measured to the nearest 0.1 kg. This was followed by an assessment of aerobic fitness. During their second visit, participants performed either a moderate intensity, continuous cycling exercise (MICE) or a high intensity, intermittent cycling exercise (HIIE). During their third visit, participants were asked to perform the exercise that they did not complete in visit #2. The order of exercises were selected in a randomized, counterbalanced fashion, such that half of the participants in the JIA and healthy control groups performed MICE followed by HIIE, while the other half performed HIIE followed by MICE. Blood samples were collected to assess EPC and CEC levels at rest, mid- and immediately post-exercise. Participants were then provided with an accelerometer to wear over a 9-day period to assess levels of moderate-to-vigorous physical activity (MVPA).

### Aerobic fitness assessment

Aerobic fitness was assessed using the McMaster All Out Continuous Progressive test on either a mechanically or electromagnetically braked cycle ergometer (Flesich-Metabo, Geneva, Switzerland; Lode Corival, Lode, The Netherlands, respectively). Progression in this test was achieved by fixed increases in workload every 2-min, such that the participant’s maximal workload was achieved by 8- to 12-min. The test was terminated when the participant was no longer able to maintain the prescribed pedaling cadence of 60-70 rpm, despite strong verbal encouragement. To assess aerobic fitness, defined here as the maximal volume of oxygen consumed over 30-sec ($\dot{\mathrm{V}}{\mathrm{O}}_{2\mathrm{peak}}$), participants were asked to breathe into a mouthpiece connected to a calibrated metabolic cart (Care Fusion, Cardinal Health) for determination of breath-by-breath inspired O_2_ and expired CO_2_. Maximal workload, defined as the peak power (Ẇ_peak_) achieved during the test prorated to the time completed in the last stage, was also determined so as to normalize the workload for the subsequent cycling tasks.

### MICE and HIIE protocols

During visits 2 and 3, participants performed either the MICE or HIIE protocols on the same cycle ergometer used in their aerobic fitness assessment. These sessions were performed at the same time of day, at least 4 days apart. The MICE protocol consisted of 2 × 30-min bouts of cycling at 50 % of Ẇ_peak_, with a 6-min rest between bouts. The continuous nature of this exercise was designed to mimic traditional adult-based exercise prescriptions that have commonly been utilized in the JIA population [35, 36]. The HIIE protocol consisted of 6 sets of 4 × 15-sec bouts of cycling at 100 % of Ẇ_peak_ for a total of 6-min of exercise; participants were given a 1-min rest between bouts, and 6-min rest between sets. The intermittent, but intense, nature of this exercise was selected to reflect the typical physical activity patterns of children [37].

### Physical activity assessment

Each participant was outfitted with an ActiGraph GT1M accelerometer (The ActiGraph, Pensacola, FL), which is a small device that provides objective and valid measures of habitual physical activity in youth [38]. Accelerometers were initialized to sample data in 3-sec intervals. Participants were instructed to wear the device over the right hip during all waking hours, with the exception of water activities, for 9 consecutive days. Levels of MVPA were determined and reported in minutes per day and minutes per hour of monitoring time, as previously described [26, 38]. Participants were included in the analyses if they wore the device for ≥ 10 h on ≥ 4 days, including 1 weekend day.

### Blood samples

Participants were instructed to avoid engaging in any strenuous activity for at least 24 h, and to refrain from eating and drinking, with the exception of water, for 3 h prior to arrival to the laboratory. Blood samples during both MICE and HIIE protocols were collected at rest approximately 15 min before exercise (REST), at the mid point of exercise (MID), and immediately at the end of exercise (POST) from an indwelling catheter placed in the ante-cubital region of the arm. For each blood sampling time point, 12 mL of whole blood was collected of which 2 mL were processed for a complete blood count by the McMaster Core Facility, and the remainder was stained for determination of EPCs and CECs, as previously described [26, 39]. Briefly, peripheral blood mononuclear cells (PBMCs) were isolated by density gradient centrifugation according to manufacturer protocols (Histopaque 1077, Sigma-Aldrich). Samples were then incubated with an FcR blocking reagent so as to minimize non-specific, receptor-mediated antibody binding. This was followed by a 20-min incubation with antibodies and conjugated fluorochromes for CD31-FITC, CD34-APC, CD45-PerCP and CD133-PE. Cells were then lysed and fixed prior to analysis for EPCs, defined as CD31^+^CD45^dim^CD34^br^CD133^+^, and CECs, identified as CD31^br^CD45^−^CD34^dim^CD133^−^ [26, 39]. Samples were analyzed within 24 h of collection on either a BD LSRII flow cytometer (2 JIA and 2 matched controls) or a Miltenyi Biotec MACSQuant flow cytometer. Pilot data from samples assessed on both units revealed no significant differences in EPC and CEC counts (Obeid et al., unpublished observation). All analyses were performed using FlowJo (Version 8.7 for MacIntosh, Treestar Inc.), and both EPC and CEC levels are reported as a concentration as well as a percentage of PBMCs.

### Statistical analyses

All variables were assessed for normality using the Shapiro-Wilk test. Independent sample t-tests were used to assess differences in anthropometric, fitness, and physical activity variables between groups. To examine differences in resting EPCs and CECs, two-way ANOVAs were performed with group (JIA vs. control) and day (Visit 2 vs. Visit 3) as factors. The effects of exercise on EPCs and CECs were compared in JIA and healthy controls by two-way ANOVA, with time (REST vs. MID vs. POST) and group (JIA vs. controls) as factors, for MICE and HIIE separately. Given the previously reported associations between EPCs, CECs, and physical activity, ANOVAs were repeated with levels of MVPA included as a covariate. Two-way ANCOVAs were also performed using resting EPC and CEC concentrations as a covariate. Significant main effects and interactions were further examined using Tukey’s HSD post hoc, with statistical significance set at p ≤ 0.05. Cohen’s *d* (ES) was calculated as a measure of effect size, standardized to the variance in the control group, where small, medium, and large effects were defined as 0.2–0.49, 0.5–0.79, and >0.79, respectively [40]. All statistical analyses were performed in Statistica (version 10.0, Statsoft, Inc., Tulsa, OK). Data are presented as mean ± SD and 95 % confidence intervals, unless otherwise specified.

## Results

### Participants characteristics

Seven patients with JIA and 6 healthy controls completed this study. Disease-related participant characteristics are presented in Table 1. Participant characteristics, fitness, and physical activity are compared in children with JIA and healthy controls in Table 2. $\dot{\mathrm{V}}{\mathrm{O}}_{2\mathrm{peak}}$, a measure of aerobic fitness, tended to be lower in JIA vs. controls, but W_peak_ was similar. There were also no differences between groups in average time spent in MVPA per day or per hour.

**Table 1:** Disease-related characteristics of children with JIA

	Age (sex)	JIA type	Disease duration (years)	CRP	ESR	Active joints	Medications
I	12.8 (F)	Psoriatic	3.8	0.34	6.0	Wrists	NSAID
II	10.8 (M)	Systemic	6.0	0.05^a^	n/a	None	None
III	17.4 (M)	Oligoarthritis (ANA-, RF+)	14.2	0.75^a^	6.0	None	None
IV	13.8 (M)	Polyarthritis (ANA-, RF-)	3.6	2.80	11.0	None	Methotrexate
V	11.1 (F)	Polyarthritis (ANA-, RF+)	3.6	0.90	15.0	Ankles	Methotrexate, Corticosteroid
VI	11.5 (F)	Polyarthritis (ANA-, RF-)	7.3	<0.20	9.0	None	Methotrexate
VII	16.5 (F)	Oligoarthritis (ANA+, RF-)	15.2	n/a	n/a	None	NSAID

*ANA* Antinuclear Antibody, *CRP* C-Reactive Protein, *ESR* Erythrocyte Sedimentation Rate, *n/a* not available, *NSAID* Non-Steroidal Anti-Inflammatory Drugs, *RF* Rheumatoid Factor. Disease duration was calculated as the date of confirmed diagnosis of arthritis to date of enrollment in the study. CRP values were based on a blood sample taken at the clinic visit closest to study participation (^a^ CRP was measured from a study visit blood sample since participants did not have any recent clinic visits). Active Joints refer to joints assessed as tender or swollen by the participant’s rheumatologist at the clinic visit closest to study participation. Medications refer to those taken regularly by the participant at the time of study completion (Off medication duration: II = 5.0 years, and III = 0.9 years)

**Table 2:** Participant characteristics by group

	JIA	Control	Mean difference (Lower, Upper 95 % CI)	P value	Effect size
N (Males)	7 (3)	6 (1)	n/a	n/a	n/a
Age (years)	13.4 ± 2.6	14.0 ± 2.3	−0.6 (-3.8, 2.6)	0.67	0.26
Height (cm)	157.5 ± 15.0	162.1 ± 5.2	−4.6 (-20.4, 11.1)	0.53	0.88
Weight (kg)	56.0 ± 16.7	50.1 ± 7.1	5.9 (-11.9, 23.8)	0.48	0.83
$\dot{V}{O}_{2\mathrm{peak}}$ (ml/kg/min)	45.6 ± 11.4	57.9 ± 8.3	−12.4 (-25.7, 1.0)	0.07	1.48
*W*_peak_ (W/kg)	3.0 ± 1.0	3.5 ± 0.5	−0.4 (-1.5, 0.7)	0.43	1.00
MVPA (min/d)	35.7 ± 13.0	26.1 ± 9.7	9.6 (-6.3, 25.5)	0.21	0.99
MVPA (min/hr wear time)	4.4 ± 2.0	3.8 ± 1.9	0.6 (-2.0, 3.3)	0.61	0.32

$\dot{\mathrm{V}}{\mathrm{O}}_{2\mathrm{peak}}$ maximal volume of oxygen consumed over 30-sec during the aerobic fitness test, *W*_*peak*_ peak workload achieved during the aerobic fitness test, *MVPA* moderate-to-vigorous physical activity. Mean difference calculated as JIA – control. Statistical significance set at p ≤ 0.05

### Resting EPCs and CECs

No differences were observed in resting levels of EPCs or CECs in youth with JIA compared with healthy controls. This finding was consistent when cells were expressed as either a proportion of PBMCs or as a concentration (p = 0.18–0.94). Mean resting EPC and CEC values by group are presented in Table 3.

**Table 3:** Resting EPC and CEC concentrations in JIA (*n* = 7) and Controls (*n* = 5)

	JIA		Controls		Effect size
	MICE	HIIE	MICE	HIIE	
EPC					
% of PBMCs	0.02 ± 0.02	0.02 ± 0.02	0.03 ± 0.02	0.02 ± 0.01	0.25
	(0.004, 0.03)	(0.003, 0.04)	(0.01, 0.05)	(0.003, 0.05)	
× 10^6^ cells/L	0.55 ± 0.48	0.55 ± 0.50	0.91 ± 0.55	0.79 ± 0.42	0.55
	(0.10, 0.99)	(0.03, 1.08)	(0.22, 1.60)	(0.24, 1.83)	
CEC					
% of PBMCs	0.23 ± 0.26	0.11 ± 0.13	0.20 ± 0.23	0.22 ± 0.22	0.17
	(0.01, 0.46)	(0.03, 0.24)	(0.09, 0.48)	(0.33, 0.77)	
× 10^6^ cells/L	6.36 ± 9.61	2.85 ± 3.19	6.28 ± 7.24	8.26 ± 8.88	0.37
	(2.52, 15.24)	(0.49, 6.20)	(2.71, 15.28)	(13.81, 30.3)	

Data are presented as mean ± SD (lower, upper 95 % CI). Effect size calculated in JIA vs. controls, standardized to the pooled SD of the control group

### Exercise responses in JIA and healthy controls

**Fig. 1:**
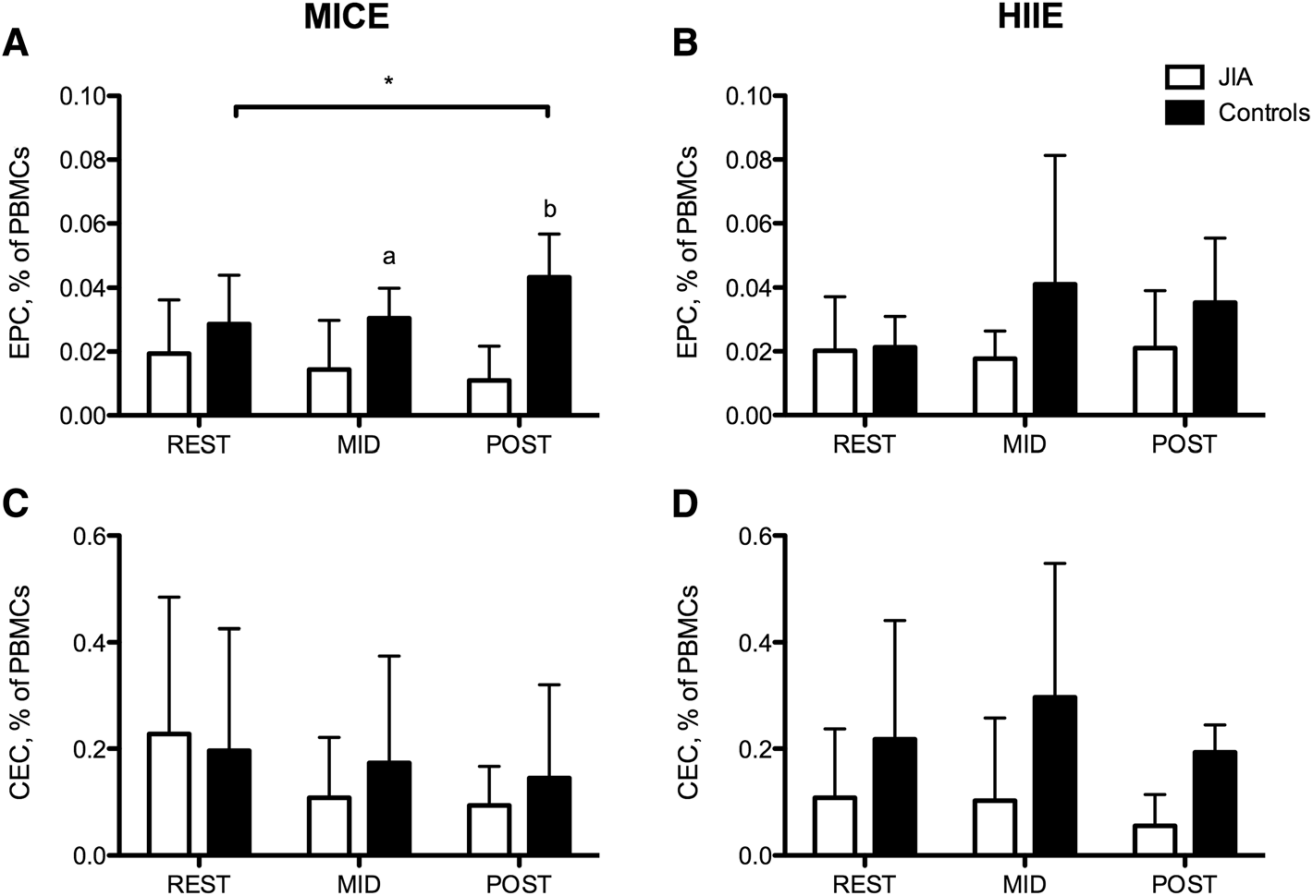
REST, MID- and POST-exercise proportions EPCs and CECs in youth with JIA (n = 7) and healthy controls (n = 5). A significant interaction was seen for EPCs as a proportion of PBMCs during MICE (**a**), whereby healthy controls demonstrated an increase in EPCs while EPCs in JIA remained unchanged. Conversely, HIIE (**b**) did not alter EPCs in healthy controls or JIA. Similarly, neither MICE (**c**) nor HIIE (**d**) had any effect on CECs in JIA and healthy controls. * significant difference from REST, p = 0.05; ^a^ significant difference between JIA and healthy controls, p = 0.05; ^b^ significant difference between JIA and healthy controls, p <0.01

While the MICE protocol led to a significant post-exercise increase in the concentration of EPCs in healthy controls, these cells remained unaltered in JIA (group × time F(2, 20) = 5.48, p = 0.01). Both MID (1.11 ± 0.39 × 10^6^ cells/L) and POST (1.68 ± 0.33 × 10^6^ cells/L) EPCs were significantly higher in healthy controls compared with REST (ES = 1.44, 2.71), MID (0.48 ± 0.50 × 10^6^ cells/L, p = 0.02–0.04; ES = 1.60, 2.89) and POST (0.43 ± 0.33 × 10^6^ cells/L, p < 0.01; ES = 1.86, 3.17) EPCs in JIA. A similar interaction was observed when EPCs were expressed as a proportion of PBMCs (F(2,20) = 5.11, p = 0.01; ES = 0.60-2.41; Fig. 1). No changes were detected in proportions or concentrations of EPCs in response to the HIIE protocol in either JIA or healthy controls (p = 0.33–0.38; ES = 0.22–1.13). Moreover, neither the MICE nor HIIE protocol had an effect on proportions or concentrations of CECs in JIA and healthy controls, as presented in Fig. 1 (p = 0.28–0.69; ES = 0.08–4.1). These findings were unchanged when resting concentrations and/or MVPA were included as covariates in the analyses.

## Discussion

This is the first study to compare exercise-induced changes in EPCs and CECs in children with JIA and healthy controls. Our findings suggest that similar resting concentrations and proportions of EPCs and CECs are present in both groups. During MICE, healthy controls demonstrated a steady increase in EPCs, which was not observed in participants with JIA. Conversely, CECs were unaltered in both groups during MICE, and HIIE had no effect on either CECs or EPCs.

Resting EPC and CEC concentrations were similar in JIA and controls, which falls in line with the recently reported findings of Rusak et al. in JIA, but differs from previous studies that showed altered EPC and CEC levels in children and adults with chronic medical conditions compared with controls [14–17, 20, 41–45]. This may be related to the fact that our participants with JIA had a small number of active joints and relatively low C-reactive protein (CRP) and erythrocyte sedimentation rates (ESR), which may suggest they also had lower levels of disease activity at the time of participation. Data from adults with RA lend additional support to this finding. More specifically, a comparison of adults with active RA (disease activity score of ≥ 3.2), low or inactive RA and healthy controls revealed that only those with active disease demonstrated significantly impaired EPCs (active: 0.026 ± 0.002 % vs. no/low active: 0.052 ± 0.006 % vs. controls: 0.045 ± 0.008 %) [14]. The authors also reported a significant, negative relationship between EPCs and disease activity score (r = -0.38, p < 0.01) [14]. Similarly, Egan et al. reported significant negative associations between EPCs and ESR as well as rheumatoid factor level, as indicators of inflammation [15]. While the only study to assess CECs in adults with RA reported higher concentrations of these cells compared with controls, the 2-fold difference between groups was less than expected when compared with other disease groups (systemic lupus erythematosus and vasculitis) that demonstrate 6- to 20-fold higher levels of CECs relative to controls [10]. The authors reasoned that this was likely related to low disease activity and drug management in the RA group [10].

The exact underlying mechanism linking EPCs, CECs, and disease activity remains unclear but is likely mediated, at least in part, by systemic levels of inflammation and medication use. Grisar et al. reported that patients with high levels of tumor necrosis factor alpha (TNF-α) presented with low EPCs, and when examined according to medication use, those on anti-TNF therapy demonstrated similar levels of EPCs to healthy controls [14]. More recently, Rusak et al. found that patients with JIA treated with a combination of glucocorticoids, methotrexate, and anti-TNF therapy presented with reduced EPCs compared with those treated with either methotrexate alone, a combination of methotrexate and glucocorticoids, and healthy controls [20]. The pilot nature of this study did not allow us to examine these factors; however, it may be important to consider both factors in future study designs.

Exercise is a potent stimulus to transiently increase EPCs in adults, whereby a single bout can lead to a 66–309 % increase in peripheral blood concentrations of these cells [25]. In children, only two studies have examined the effect of an acute bout of exercise on EPCs [23, 46]. Zaldivar et al. assessed healthy, pre- and post-pubertal males and reported an 83–170 % increase in EPCs [23]. Conversely, Lau et al. examined youth with chronic kidney disease and reported no change in EPCs [46]. In the present study, a single bout of moderate intensity, continuous exercise led to a ~100 % increase in EPCs in healthy children, but did not elicit any change in EPCs in participants with JIA. The fact that we were unable to detect a change in EPCs in JIA may suggest an impaired mobilization of EPCs from the bone marrow. In healthy individuals, a number of angiogenic factors are associated with increased EPC proliferation and mobilization into the circulation; chief among these is Vascular Endothelial Growth Factor (VEGF) [47]. Interestingly, elevated VEGF levels are consistently reported in RA, but are not matched by an increase in EPCs [14, 41, 43, 47]. The mechanisms inhibiting the commonly reported effects of VEGF on EPCs in RA remain unknown. If our participants presented with chronically elevated VEGF, it is plausible that the exercise stimulus may not have been sufficient to stimulate additional VEGF production to promote EPC mobilization; however, this cannot be ascertained since VEGF levels were not assessed. Alternatively, we may have failed to detect a change in peripheral blood EPCs if they were rapidly taken up by another tissue. In fact, there is some evidence to suggest that EPCs may be recruited from the peripheral blood into the synovium in RA [48]. It has been hypothesized that EPCs may be trapped in the synovium leading to increased synovial blood vessel formation, recruitment of inflammatory markers to the affected joints, and a reduced ability for EPCs to respond to CV damage [43, 48, 49]. Since EPCs were similar in JIA and healthy controls at rest, it seems unlikely that an acute bout of exercise would lead to a substantial increase in EPC recruitment to the synovium. However, we cannot rule out the possibility that the timing of our blood samples may have limited our ability to detect changes in EPCs in JIA. Taken together, our data suggest that exercise affects EPCs levels differently in youth with JIA and healthy controls.

Given that short bouts of intense exercise led to significant increases in EPCs in the work of Zaldivar et al., we anticipated similar increases with our HIIE protocol [23]. However, we did not detect any changes in EPCs in either JIA or healthy controls during HIIE. This finding may be related to the timing of our blood collection (too early, or too late), which may have missed a peak in EPCs. It is more likely that the exercise duration of our HIIE protocol was not sufficient to alter levels of these cells. In fact, when EPC responses in adults were compared following 30-min of high intensity, 30-min of moderate intensity, or 10-min of moderate intensity running, only the 30-min bouts led to similar increases in EPCs [22].

Neither HIIE nor MICE had any effect on CEC concentrations in participants with JIA or healthy controls. Given that CECs are a population mature endothelial cells detached from the vasculature following irreparable damage, they are likely more representative of long-term dysfunction and cumulative injury to the endothelium [10]. It is plausible that a single, acute bout of exercise, regardless of intensity or duration, may not be sufficient to induce any changes in CEC concentrations. Whether repeated bouts of exercise can modify CEC concentrations remains to be determined.

A number of limitations should be considered in the interpretation of our findings. First, this study assessed a small group of patients with different types of JIA making it difficult to determine the generalizability of our results. However, the majority of the calculated effect sizes were medium or large, suggesting the reported results were meaningful, irrespective of sample size. Second, there is no consensus with respect to the best protocol and markers for EPC and CEC enumeration [18, 39, 50]. Because of similarities in cell surface markers, there is a chance that some mature endothelial cells and hematopoietic stem cells were gated with EPCs and vice versa. Despite this potential overlap, the high degree of repeatability and reliability of our EPC (intraclass correlation coefficient, ICC = 0.92 and 0.98, respectively) and CEC (ICC = 0.96 and 0.82) enumeration protocol lends support to the sensitivity of our measurements (Obeid, unpublished observation). Third, our EPCs and CECs response profile is limited to 2 time points at MID- and POST-exercise, which may not reflect the peak response times of these cells. Finally, we assessed EPC and CEC concentrations but did not assess their function in the circulation. Future work should seek to include functional assays given that there may be a disconnect in the quantity and function of EPCs in adults with chronic conditions [41, 47, 49].

## Conclusion

To our knowledge, this was the first study to simultaneously compare EPCs and CECs at rest and in response to exercise in youth with JIA and healthy controls. Our results suggest that resting levels of both EPCs and CECs are similar in JIA and healthy controls. In addition, EPCs in healthy controls increased by an average of ~100 % in response to an acute bout of moderate intensity, continuous cycling but remained unchanged in JIA. Conversely, high intensity, intermittent exercise had no effect on either EPCs or CECs in JIA and controls. The EPC response to exercise in JIA may be blunted as a result of disease-related inflammation and medication use, but may also reflect recruitment of EPCs from the circulation into the synovium. Future studies should explore factors that may be inhibiting exercise-induced changes in EPCs, as well as the impact of blood sample timing on EPC and CEC responses to exercise. Given the growing body of evidence supporting the role of these cells in maintaining and improving vascular health, a better understanding of the effects of exercise on their concentrations and function may allow for development of an appropriate adjuvant therapy to maintain CV health in JIA.

## Abbreviations

ANA: Antinuclear antibody
ANOVA: Analysis of variance
CEC: Circulating endothelial cell
CI: Confidence interval
CRP: C-reactive protein
CV: Cardiovascular
CVD: Cardiovascular disease
EPC: Endothelial progenitor cell
ES: Effect size
ESR: Erythrocyte sedminentation rate
HIIE: High intensity, intermittent exercise
ICC: Intraclass correlation coefficient
JIA: Juvenile idiopathic arthritis
MICE: Moderate intensity, continuous exercise
MID: Mid-point of exercise
MVPA: Moderate-to-vigorous physical activity
NSAID: Non-steroidal anti-inflammatory drugs
PBMC: Peripheral blood mononuclear cells
POST: Immediately post-exercise
RA: Rheumatoid arthritis
REST: At rest, pre-exercise
RF: Rheumatoid factor
VEGF: Vascular endothelial growth factor
$\dot{\mathrm{V}}{\mathrm{O}}_{2\mathrm{peak}}$: Maximal volume of oxygen consumed
Ẇ_peak_: Peak mechanical power
